# Cisplatin Resistance in Osteosarcoma: *In vitro* Validation of Candidate DNA Repair-Related Therapeutic Targets and Drugs for Tailored Treatments

**DOI:** 10.3389/fonc.2020.00331

**Published:** 2020-03-10

**Authors:** Marilù Fanelli, Elisa Tavanti, Maria Pia Patrizio, Serena Vella, Amira Fernandez-Ramos, Federica Magagnoli, Silvia Luppi, Claudia Maria Hattinger, Massimo Serra

**Affiliations:** IRCCS Istituto Ortopedico Rizzoli, Laboratory of Experimental Oncology, Pharmacogenomics and Pharmacogenetics Research Unit, Bologna, Italy

**Keywords:** osteosarcoma, DNA repair, cisplatin, drug resistance, chemotherapy, targeted drugs, tailored treatment

## Abstract

Treatment of high-grade osteosarcoma, the most common malignant tumor of bone, is largely based on administration of cisplatin and other DNA damaging drugs. Altered DNA repair mechanisms may thus significantly impact on either response or resistance to chemotherapy. In this study, by using a panel of human osteosarcoma cell lines, either sensitive or resistant to cisplatin, we assessed the value as candidate therapeutic targets of DNA repair-related factors belonging to the nucleotide excision repair (NER) or base excision repair (BER) pathways, as well as of a group of 18 kinases, which expression was higher in cisplatin-resistant variants compared to their parental cell lines and may be indirectly involved in DNA repair. The causal involvement of these factors in cisplatin resistance of human osteosarcoma cells was validated through gene silencing approaches and *in vitro* reversal of CDDP resistance. This approach highlighted a subgroup of genes, which value as promising candidate therapeutic targets was further confirmed by protein expression analyses. The *in vitro* activity of 15 inhibitor drugs against either these genes or their pathways was then analyzed, in order to identify the most active ones in terms of inherent activity and ability to overcome cisplatin resistance. NSC130813 (NERI02; F06) and triptolide, both targeting NER factors, proved to be the two most active agents, without evidence of cross-resistance with cisplatin. Combined *in vitro* treatments showed that NSC130813 and triptolide, when administered together with cisplatin, were able to improve its efficacy in both drug-sensitive and resistant osteosarcoma cells. This evidence may indicate an interesting therapeutic future option for treatment of osteosarcoma patients who present reduced responsiveness to cisplatin, even if possible effects of additive collateral toxicities must be carefully considered. Moreover, our study also showed that targeting protein kinases belonging to the mitogen-activated protein kinase (MAPK) or fibroblast growth factor receptor (FGFR) pathways might indicate new promising therapeutic perspectives in osteosarcoma, demanding for additional investigation.

## Introduction

Osteosarcoma (OS) is the most common malignant tumor of bone, which accounts for about 5% of childhood and adolescence neoplasms. High-grade OS is usually treated with neoadjuvant chemotherapy protocols based on cisplatin (CDDP), doxorubicin, methotrexate, and ifosfamide. However, despite this aggressive approach, 35–45% of patients still recur and experience an unfavorable outcome (1–5).

Three out of the four conventional drugs, which are most commonly used in first-line chemotherapy for high-grade OS, induce DNA damages either directly (CDDP and ifosfamide) or indirectly (doxorubicin). Therefore, resistance mechanisms related to DNA damage response can significantly impact on OS chemotherapy unresponsiveness. Among these drugs, CDDP is the agent which has most extensively been studied in relation to DNA repair. A consistent body of evidence is showing that the onset of clinical unresponsiveness to CDDP usually creates further therapeutic complications, because patients can also become cross-resistant to the other DNA damaging chemotherapeutic drugs used in first- or rescue treatment protocols (4, 6).

One of the most important mechanisms of resistance against CDDP is repair of drug-induced DNA damages via different pathways, of which the most common is the nucleotide excision repair (NER) (4, 7, 8). We have recently obtained data indicating that protein overexpression of the NER gene *ERCC excision repair 1 (ERCC1)* negatively impacts on the clinical responsiveness to CDDP-based treatments and on patients' outcome (9). However, knowledge about the relevance of both *ERCC1* and other DNA repair genes for resistance to CDDP and DNA damaging drugs in OS still needs to be implemented.

In addition to NER, other DNA repair pathways, first of all the base excision repair (BER), have been indicated or proved to be implicated in CDDP resistance of several human tumors (10–12), but their relative impact significantly varies among different neoplasms and only very few information is available for OS (4).

Cellular response to CDDP-induced DNA damage is also mediated by downstream effects on cell cycle and mitosis regulation (7, 11). The interplay between DNA damage response and the proliferation machinery is based on the activity of several protein kinases, which in some tumors have been demonstrated to be involved in CDDP resistance (13). In human OS cells, we have obtained evidence of a possible involvement of aurora kinases in CDDP resistance (14) and of cyclin-dependent kinases (CDKs) in repair of CDDP-induced DNA damages (15), but this field of research still remains open.

Based on our previously (unpublished) gene expression analyses, we observed that CDDP-resistant human OS cell lines showed increased expression of several kinases in comparison with their corresponding parental cells. Among these kinases, 18 can be targeted by inhibitor drugs of which some have already entered clinical trials or have shown promising preclinical activities in human cancers different from OS.

In this study, we first confirmed the expression level of these 18 kinases in human OS CDDP-resistant variants in comparison with their parental cell lines.

Moreover, the role of genes belonging to NER or BER pathways and of the aforementioned 18 kinases for CDDP resistance in human OS cells was estimated, in order to indicate new candidate markers, which may be considered to overcome resistance to CDDP in OS patients.

Finally, the *in vitro* efficacy of drugs targeting the most significantly emerged genes or pathways has been assessed.

## Materials and Methods

### Experimental Models

The *in vitro* studies were performed on the U-2OS and Saos-2 human OS cell lines and a panel of variants resistant to CDDP (U-2OS/CDDP300; U-2OS/CDDP1 μg; U-2OS/CDDP4 μg; Saos-2/CDDP300; Saos-2/CDDP1 μg; Saos-2/CDDP6 μg).

The U-2OS and Saos-2 cell lines were purchased from the American Type Culture Collection (ATCC, Rockville, MD). Variants resistant to CDDP were established by exposing the drug-sensitive U-2OS and Saos-2 parental cell lines to stepwise increasing concentrations of CDDP and characterized as previously described (16).

DNA fingerprint analyses of 17 polymorphic short tandem repeat sequences were performed for all cell lines, confirming their identity.

All cell lines were cultured in Iscove's modified Dulbecco's medium (IMDM), supplemented with penicillin (20 U/ml)/streptomycin (20 U/ml) (Invitrogen Ltd., Paisley, UK) and 10% heat-inactivated fetal bovine serum (FBS; Biowhittaker Europe, Cambrex-Verviers, Belgium), and maintained at 37°C in a humidified 5% CO_2_ atmosphere. Drug resistant variants were continuously cultured in presence of CDDP at the concentration used for their selection.

### Gene Expression Analyses

Analyses focused on genes belonging to the NER and BER pathways, which are known to play key roles for CDDP resistance in several human cancers, and on the 18 druggable protein kinases selected on the basis of our previous observations, which indicated their increased expression in U-2OS- and/or Saos-2-derived CDDP-resistant variants in comparison with their parental cells (Table 1). Expression level of these genes was assessed by quantitative reverse transcriptase-polymerase chain reaction (qRT-PCR), in order to confirm their overexpression in CDDP-resistant variants compared to their parental cell lines. For each gene, 500 ng of total RNA were reverse transcribed using the High Capacity cDNA Archive Kit (Applied Biosystems, Foster City, CA) according to the manufacturer's protocol. cDNAs were aliquoted and stored at −20°C until use. To quantify the fold-change in gene expression, the TaqMan Gene Expression Assays listed in Supplementary Table 1 were used on the ViiA 7 instrument (Applied Biosystems). GAPDH (Assay Hs99999905_m1; Applied Biosystem) was used as reference gene.

**Table 1:** DNA repair and kinase genes analyzed in this study.

**Pathway/family**	**Name (Gene ID)**	**Full gene name**
Nucleotide excision repair (NER)	*ERCC1* (2067)	ERCC excision repair 1
	*ERCC2/XPD* (2068)	ERCC excision repair 2/Xeroderma pigmentosum D
	*ERCC3/XPB* (2071)	ERCC excision repair 3/Xeroderma pigmentosum B
	*ERCC4/XPF* (2072)	ERCC excision repair 4/Xeroderma pigmentosum F
	*ERCC5/XPG* (2073)	ERCC excision repair 5/Xeroderma pigmentosum G
	*XPA* (7507)	Xeroderma pigmentosum A
Base excision repair (BER)	*PARP1* (142)	poly(ADP-ribose) polymerase 1
	*PARP2* (10038)	poly(ADP-ribose) polymerase 2
Kinases	*AKT3* (10000)	AKT serine/threonine kinase 3
	*CDK3* (1018)	Cyclin dependent kinase 3
	*CDK6* (1021)	Cyclin dependent kinase 6
	*CDK8* (1024)	Cyclin dependent kinase 8
	*CDK9* (1025)	Cyclin dependent kinase 9
	*CDK10* (8558)	Cyclin dependent kinase 10
	*FGFR1* (2260)	Fibroblast growth factor receptor 1
	*FGFR2* (2263)	Fibroblast growth factor receptor 2
	*FLT4* (2324)	Fms related tyrosine kinase 4
	*MAP2K2* (5605)	Mitogen-activated protein kinase kinase 2
	*MAP2K3* (5606)	Mitogen-activated protein kinase kinase 3
	*MAP2K5* (5607)	Mitogen-activated protein kinase kinase 5
	*MAP2K7* (5609)	Mitogen-activated protein kinase kinase 7
	*MAPK1* (5594)	Mitogen-activated protein kinase 1
	*MAPK3* (5595)	Mitogen-activated protein kinase 3
	*PIK3C2A* (5286)	Phosphatidylinositol-4-phosphate 3-kinase catalytic subunit type 2 alpha
	*PIK3C3* (5289)	Phosphatidylinositol 3-kinase catalytic subunit type 3
	*PIK3CB* (5291)	Phosphatidylinositol-4,5-bisphosphate 3-kinase catalytic subunit beta

### Gene Silencing

In a first set of experiments, each gene was silenced by transfecting cells with three different siRNAs specific for different regions of the same gene (customized Ambion Silencer Select siRNAs library, purchased from Thermo Fisher Scientific, Waltham, MA) for 24 h, whereas controls were cultured in presence of scrambled siRNAs. Transfection was performed by using each siRNA at a final concentration of 5 nM and 0.3 or 1.25 μl lipofectamin RNAiMAX (Invitrogen, Thermo Fisher Scientific) per well in a 96-well or 24-well plate according to the manufacturer's protocol. After 24 h, medium was changed and cells were maintained in siRNA-free medium for additional 48 h. The extent of gene silencing was estimated at 72 h for each siRNA by qRT-PCR on the ViiA 7 instrument (Thermo Fisher Scientific) in order to identify the siRNA with the strongest effect on mRNA down-regulation. Gene expression analysis was performed using the TaqMan® Gene Expression Cells-to-CT™ Kit (Invitrogen, Thermo Fisher Scientific) and appropriate TaqMan® Gene Expression Assays (Applied Biosystems) listed in Supplementary Table 1. GAPDH (Assay ID:Hs99999905_m1; Applied Biosystem) was used as reference gene.

The siRNAs producing the highest mRNA down-regulation were then selected to verify whether the inhibition of a specific gene expression was related to a corresponding increase in CDDP sensitivity. For this second set of experiments, 48 h after seeding and transfection, the cells were incubated with different dosages of CDDP for additional 48 h. Controls were incubated with scrambled siRNAs. The *in vitro* sensitivity to CDDP was estimated on the basis of drug dosage response curves, assessed by using the 3-(4,5-dimethylthiazol-2-yl)-2,5-dephenyltetrazolium bromide (MTT) assay kit (TACS MTT Cell Proliferation Assay, Trevigen, Gaithersburg, MD). For all cell lines, the IC50 value (CDDP concentration inducing 50% growth inhibition) was determined inside each experimental condition. To quantify the extent of the increased CDDP sensitivity after gene knock-down, ratios between the IC50 values of cells incubated with scrambled siRNAs and those of silenced cells were calculated.

### Western Blot

Cells were cultured in petri dishes until confluence, harvested by scraping and lysed in RIPA buffer supplemented with protease and phosphatase inhibitor cocktails (Thermo Fisher Scientific) and Benzonase (Sigma-Aldrich, St. Louis, MO). Protein concentrations were determined by the Bradford Protein Assay (Bio-Rad Laboratories Italia, Segrate, Italy). Equal amounts of cell lysates (80 μg) were separated by SDS-PAGE on 4–20% gradient gels (Thermo Fisher Scientific) and then transferred onto nitrocellulose membranes (Bio-Rad Laboratories Italia, Segrate, Italy). Then, membranes were blocked in 5% BSA in 1 X TBS containing 0.1% Tween-20 (TBST; Tris-Buffered Saline and Tween 20) and incubated in primary antibodies (Supplementary Table 2) overnight at 4°C, washed in 1 X TBST and incubated with the appropriate secondary antibody (goat anti-mouse or anti-rabbit IgG-HRP, Santa Cruz Biotechnology, 1:10,000) for 1 h. Blots were washed three times with 1 X TBST, detected with the SuperSignal West Pico Reagent (Thermo Fisher Scientific, Waltham, MA), and visualized in a ChemiDoc digital imaging station (Bio-Rad).

Protein loading was assessed by coomassie R-250 staining (Bio-Rad). Fold changes in protein expression level were determined by densitometric analysis of western blots and autoradiographs using the publicly available ImageJ software (National Institutes of Health, Bethesda, MD, USA).

### Drugs

The drugs targeting the prioritized genes/pathways, which have been tested for their *in vitro* efficacy, are listed in the Supplementary Table 3. Drugs were selected on the basis of their reported promising activity in other experimental models and/or their use in clinical trials for human tumors. NSC130813 (NERI02; F06), X80 and hypothemycin were purchased from Sigma-Aldrich-Merck KGaA (Darmstadt, Germany), whereas all the other drugs were purchased from Selleckchem Europe (Munich, Germany).

### *In vitro* Drug Analyses

*In vitro* drug efficacy was assessed in terms of *in vitro* growth inhibition activity estimated with the MTT assay (as described above) on the two parental cell lines (U-2OS and Saos-2) and their CDDP-resistant variants with, respectively, the lowest and the highest resistance level (U-2OS/CDDP300, U-2OS/CDDP4 μg, Saos-2/CDDP300, and Saos-2/CDDP6 μg). For three drugs, X80, quercetin and SSR128129E, the CellTiter-FluorTM Cell Viability assay (Promega, Madison, WI) was used according to the manufacturer's protocol. For each cell line, the drug IC50 value was determined, in order to highlight the possible presence of cross-resistance due to the mechanisms present in CDDP-resistant variants.

The two most active drugs which emerged from these analyses were prioritized for further evaluations. The efficacy of the *in vitro* association of each prioritized drug with CDDP was determined after 96 h of combined treatment with the IC50 dosage of each drug. In drug sequence experiments, cell lines were sequentially exposed for 48 h to their corresponding IC50 dosage of CDDP and then to the IC50 dosage of each prioritized drug for additional 48 h. These combinations were then repeated with the opposite sequence. The type of interaction in terms of synergism, antagonism or additivity, was defined on the basis of the combination index (CI) of each two-drugs combination, which was calculated with the equation of Chou-Talalay by using the CalcuSyn software (Biosoft, Stapleford, UK). By following the CalcuSyn software indications, the drug–drug interaction was classified as synergistic (SYN) when CI was lower than 0.90, additive (ADD) when CI ranged between 0.90 and 1.10, or antagonistic (ANT) when CI was higher than 1.10.

## Results

### Gene Expression Level

Expression levels of genes listed in Table 1 were assessed by RT-PCR in U-2OS/CDDP-resistant variants and compared with those of their parental cell lines. As shown in Table 2, by considering a cut-off of at least 2.0-fold increase compared to parental cells, expression of all NER or BER genes was generally enhanced in CDDP300 and CDDP4μg resistant variants, with other level increases (1.8–1.9 fold) very closed to this cut-off value. Among kinases, those which showed evidence of a higher expression in at least two CDDP resistant variants included *CDK3, FLT4, MAP2K3, MAP2K5, MAPK1, MAPK3, PIK3C3*.

**Table 2:** Expression of NER, BER, and kinase genes considered in this study assessed by RT-PCR.

**Gene**	**fold vs. U-2OS**		
	**U2CDDP300**	**U2CDDP1ug**	**U2CDDP4ug**
*ERCC1*	2.1	1.1	3.2
*ERCC2*	2.6	1.6	2.8
*ERCC3*	2.3	1.6	1.8
*ERCC4*	2.5	0.6	2.9
*ERCC5*	1.9	0.7	3.9
*XPA*	1.9	0.4	1.8
*PARP1*	2.7	0.8	1.6
*PARP2*	3.2	0.9	1.6
*AKT3*	0.9	1.0	0.5
*CDK3*	3.3	2.2	3.3
*CDK6*	1.2	1.0	0.8
*CDK8*	1.3	1.5	1.8
*CDK9*	1.6	1.5	2.8
*CDK10*	1.3	1.5	1.1
*FGFR1*	1.4	1.5	1.0
*FGFR2*	1.2	0.3	0.2
*FLT4*	5.8	3.9	4.2
*MAP2K2*	1.9	1.8	2.0
*MAP2K3*	1.2	3.4	2.2
*MAP2K5*	1.8	2.6	6.1
*MAP2K7*	0.3	1.8	1.1
*MAPK1*	3.4	2.9	3.5
*MAPK3*	2.1	3.0	3.0
*PIK3C2A*	1.5	0.8	2.4
*PIK3C3*	3.6	2.6	4.5
*PIK3CB*	1.3	1.5	1.8

*Table shows the fold-changes in the U-2OS/CDDP-resistant variants referred to its parental cell line. Highlighted values indicate fold-increases ≥ 2.0*.

### Screening and Selection of the Most Active siRNAs

RNA interference was used to determine the causal involvement in CDDP resistance of the genes listed in Table 1 by silencing each gene in the U-2OS parental cell line and its CDDP-resistant variants (U-2OS/CDDP300; U-2OS/CDDP1 μg; U-2OS/CDDP4 μg). The most effective siRNAs were identified through an extensive RNA interference approach, in which each gene was silenced by using three different siRNAs. All the selected siRNAs (Supplementary Table 4) proved to efficiently down-regulate the expression of their target genes and were used for the next phases of the study.

### Reversal of CDDP Resistance After Gene Silencing

Cell lines silenced with the siRNAs listed in the Supplementary Table 4 and their related controls were treated with CDDP, in order to verify whether a specific gene down-regulation was associated with a corresponding increase of the *in vitro* CDDP sensitivity. Table 3 shows the fold-changes in CDDP IC50 after gene silencing. As specified in the Materials and Methods section, these values represent the ratio between the CDDP-IC50 between cells incubated with scrambled siRNAs (controls) and those of silenced cells and, therefore, they reflect the increased sensitivity to CDDP consequent to each gene knock-down. By considering ratios > 2.0, results can be summarized as follows:

- Silencing of *ERCC1, ERCC2/XPD, ERCC3/XPB, ERCC4/XPF*, and *XPA* increased CDDP sensitivity in the U-2OS/CDDP300 and U-2OS/CDDP1 μg resistant variants. Silencing of *ERCC1, ERCC2/XPD*, and *ERCC4/XPF* increased CDDP sensitivity also in U-2OS parental cells
- *FGFR1* silencing increased CDDP sensitivity in the U-2OS/CDDP1 μg and in parental cells
- Silencing of *MAP2K3, MAPK3*, and *PIK3CB* was associated with an increase of CDDP sensitivity in the U-2OS/CDDP4 μg resistant variant.

**Table 3:** Fold-changes in cisplatin IC50 after gene silencing.

**Gene**	**U-2OS**	**U-2OS/CDDP300**	**U-2OS/CDDP1 μg**	**U-2OS/CDDP4 μg**
*ERCC1*	3.1	9.3	6.5	1.6
*ERCC2/XPD*	2.8	6.9	5.0	1.5
*ERCC3/XPB*	1.2	2.0	2.6	0.9
*ERCC4/XPF*	2.0	2.9	2.8	1.1
*ERCC5/XPG*	0.6	0.5	0.8	0.3
*XPA*	1.4	3.3	3.5	1.5
*PARP1*	0.3	0.6	1.0	0.7
*PARP2*	0.9	1.3	1.1	0.9
*AKT3*	1.6	1.3	0.9	1.1
*CDK3*	0.9	1.2	1.2	1.0
*CDK6*	0.8	0.9	0.9	1.4
*CDK8*	1.3	0.5	0.6	1.6
*CDK9*	1.2	0.6	1.0	1.2
*CDK10*	1.4	1.3	1.3	1.6
*FGFR1*	2.0	1.2	2.2	1.2
*FGFR2*	1.2	1.2	0.9	1.0
*FLT4*	0.8	1.0	0.9	0.8
*MAP2K2*	0.9	1.0	1.2	1.5
*MAP2K3*	0.6	1.0	1.4	2.1
*MAP2K5*	1.1	1.0	1.2	1.6
*MAP2K7*	0.9	1.0	1.3	1.5
*MAPK1*	0.6	0.8	1.2	1.1
*MAPK3*	1.3	1.0	1.3	2.6
*PIK3C2A*	0.6	0.8	1.1	1.8
*PIK3C3*	1.4	1.2	1.1	1.7
*PIK3CB*	0.8	1.4	1.2	2.3

*Values indicate ratios between the cisplatin IC50 values of silenced cells and those of cells incubated with scrambled siRNAs (controls). Highlighted values indicate ratios ≥ 2.0*.

Additional reversal activity of CDDP resistance, with IC50 ratios close to the 2.0 cut-off value, was observed for the knock-down of these and other genes (Table 3), even if this evidence was not taken into account for the candidate drug targets prioritization.

### Candidate Drug Targets Prioritization

By coupling the results derived from the assessment of gene expression level in association with CDDP resistance (Table 2) and the evaluation of increase in CDDP sensitivity after gene silencing (Table 3), the following genes were selected as candidate drug targets and were prioritized for the next phases of the study: *ERCC1, ERCC2/XPD, ERCC3/XPB, ERCC4/XPF, XPA, MAP2K3, MAPK3*, and *PIK3CB*. *FGFR1* was also selected based on its ability to reverse CDDP resistance also if its expression was found to be moderately higher than in parental cells. All these genes showed evidence of increased expression in CDDP resistant variants and their knock-down proved to be associated with an enhancement of CDDP sensitivity.

### Western Blot

**Figure 1:**
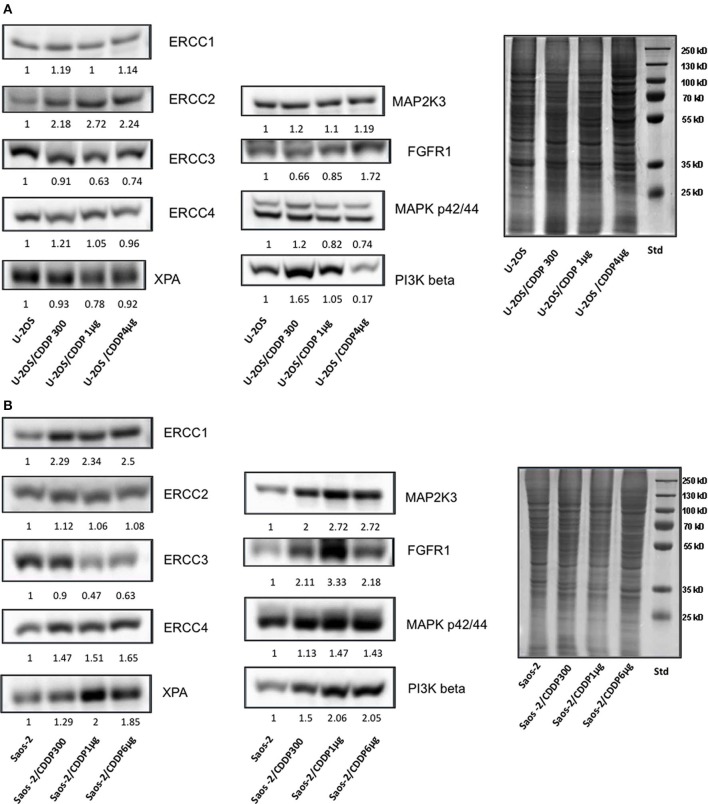
Assessment by western blot of protein expression level in CDDP-resistant variants derived from U-2OS **(A)** and Saos-2 **(B)** in relation to their corresponding parental cells.

To further validate these genes as candidate therapeutic targets to overcome CDDP-resistance, their expression at protein level was assessed by western blot in both U-2OS and Saos-2 parental cell lines and all their CDDP-resistant variants. All proteins encoded by these prioritized genes proved to be expressed in all cell lines. In the U-2OS series (Figure 1A), there was evidence of a trend toward an increased protein level in CDDP resistant variants for ERCC2, and in some variants for ERCC1, ERCC4, MAP2K3, FGFR1, and PI3K beta. In the Saos-2 series (Figure 1B), a more clear evidence of increased protein levels in CDDP resistant variants was observed for all prioritized genes, excepting ERCC2 and ERCC3.

When considered together, these results further supported the indication of all these genes as candidate drug targets.

### Efficacy of Drugs Against Selected Candidate Targets

The *in vitro* activity of drugs listed in Supplementary Table 3 was assessed by estimating their IC50 on parental cell lines (U-2OS and Saos-2) and on their resistant variants with the lowest and the highest CDDP resistance level (U-2OS/CDDP300; U-2OS/CDDP4 μg; Saos-2/CDDP300; and Saos-2/CDDP6 μg, respectively).

**Figure 2:**
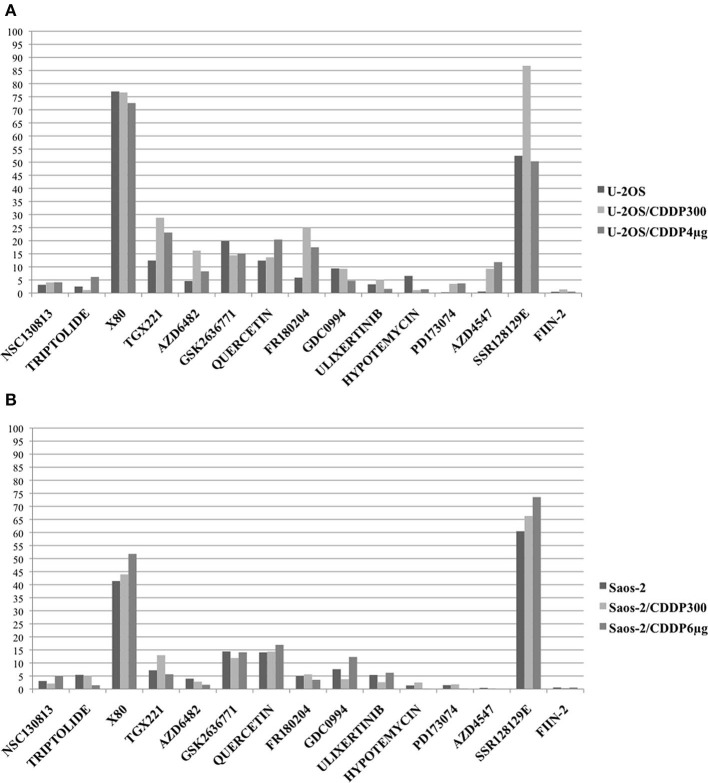
*In vitro* activity of drugs against selected target genes was assessed by estimating their IC50 on parental cell lines U-2OS **(A)** and Saos-2 **(B)** and on their CDDP resistant variants with the lowest and the highest resistance level (U-2OS/CDDP300; U-2OS/CDDP4 μg; Saos-2/CDDP300; and Saos-2/CDDP6 μg). Graphs show the IC50 values (μM) determined after 96 h of *in vitro* drug treatment (Y axis). For triptolide, IC50 values are expressed in nM.

As shown in Figure 2, several drugs showed IC50 lower than 5 μM in all cell lines. In both U-2OS and Saos-2 drug sensitive and CDDP-resistant cells, X80 and SSR128129E showed very high IC50 values, whereas TGX221, GSK2636771, quercetin, and (at a lower extent) GDC0994 showed IC50 values higher than 5 μM.

In U-2OS variants (Figure 2A), higher IC50 values in CDDP-resistant variants compared to parental cell lines (indicating possible cross-resistance) were observed for TGX221, AZD6482, FR180204, AZD4547 and, at a lower extent, for quercetin. In Saos-2 variants (Figure 2B), a possible cross-resistance with CDDP was observed for GDC0994.

By considering together the findings obtained in both U-2OS and Saos-2 cell line series, the most active DNA repair-targeting agents, without evidence of cross-resistance, proved to be NSC130813 (NERI02; F06; targeting the interaction between ERCC1 and ERCC4/XPF) and triptolide (targeting ERCC3/XPB).

The most active kinase-targeting drugs, without evidence of cross-resistance with CDDP, were ulixertinib (targeting the downstream MAPKs signaling pathway), hypotemycin (targeting the MAP2K pathway), PD173074 and FIIN-2 (both targeting FGFR1).

In order to verify whether treatment with each inhibitor drug was able to increase sensitivity to CDDP, the same group of cell lines were incubated with increasing CDDP concentrations in the absence (control) or presence of the IC20 dosage of each inhibitor drug. A ratio ≥ 2.0 (meaning a decrease of at least 2-fold of CDDP-IC50 in presence of the inhibitor drug) was considered as indication of a drug-induced CDDP sensitization.

NSC130813 (NERI02; F06) and triptolide proved to be the two drugs with the most relevant activity, being able to increase CDDP sensitivity for more than 2-fold in all the U-2OS and Saos-2 cell lines (Table 4).

**Table 4:** Fold-decrease in cisplatin (CDDP) IC50 induced by targeted drugs.

**Drug**	**U-2OS**	**U-2OS CDDP300**	**U-2OS CDDP4μg**	**Saos-2**	**Saos-2 CDDP300**	**Saos-2 CDDP6μg**
NSC130813 (NERI02, F06)	12.6	2.1	2.0	16.3	13.1	3.8
Triptolide	2.0	3.7	2.3	3.7	4.2	2.4
X80	1.78	0.8	1.2	0.9	0.9	0.8
AZD6482	1.5	1.2	1.1	2.9	2.5	1.2
GSK2636771	1.2	1.5	0.8	5.0	4.5	1.3
Quercetin	1.0	1.3	0.8	2.8	1.2	1.4
TGX221	1.5	1.2	0.8	3.0	6.9	1.3
FR180204	1.5	1.2	1.1	2.5	5.2	1.4
GDC0994	1.6	2.0	1.1	2.5	2.9	0.9
Ulixertinib (BVD-523)	1.4	1.0	1.1	1.6	0.8	1.2
Hypothemycin	0.8	0.9	0.9	2.1	0.7	1.2
AZD4547	1.2	1.2	1.0	1.2	0.7	1.2
FIIN-2	2.4	1.4	0.8	1.7	1.3	2.5
PD173074	2.1	1.2	0.8	2.5	4.1	1.2
SSR128129E	1.2	0.6	1.8	0.9	0.7	1.8

*Values indicate ratios between the CDDP IC50 of cells incubated with increasing CDDP concentrations in absence (reference) or presence of the IC20 dosage of each target inhibitor drug. Highlighted values indicate ratios ≥ 2.0. Data refer to the mean ratio value of three different experiments*.

For all these reasons, NSC130813 (NERI02; F06) and triptolide were prioritized for evaluation in combination experiments with CDDP.

### Combined Treatments

NSC130813 (NERI02; F06) and triptolide were tested in combination with CDDP, in order to verify whether these treatments lead to positive interactions. As shown in Table 5, association with CDDP produced positive (additive or synergistic) effects in both CDDP-sensitive and resistant cell lines. The only antagonistic interaction was observed in the U-2OS cell line treated with CDDP in association with NSC130813 (NERI02; F06).

**Table 5:** Interaction of NSC130813 and triptolide with cisplatin (CDDP) in drug association experiments.

**Cell line**	**CDDP + Triptolide**	**CDDP + NSC130813**
U-2OS	ADD (1.08)	ANT (1.36)
U-2OS/CDDP300	SYN (0.65)	ADD (0.97)
U-2OS/CDDP4 μg	SYN (0.59)	ADD (1.10)
Saos-2	SYN (0.41)	SYN (0.67)
Saos-2/CDDP300	SYN (0.45)	SYN (0.41)
Saos-2/CDDP6 μg	SYN (0.32)	SYN (0.71)

*Legend: Data refer to at least two different determinations. Number in parenthesis indicate the combination index (CI) values. SYN, synergistic (CI < 0.90); ADD, additive (0.90 ≤ CI ≤ 1.10); ANT, antagonistic (CI > 1.10)*.

Sequential drug exposure experiments (Table 6) mainly revealed antagonistic effects when CDDP was combined with triptolide, independently from the sequence of drug administration. Treatment with CDDP followed by NSC130813 (NERI02; F06) invariably produced antagonistic effects, whereas the opposite sequence proved to be mainly additive or synergistic (Table 6).

**Table 6:** Interaction of NSC130813 and triptolide with cisplatin (CDDP) in drug sequence experiments.

	**Treatment schedule**			
**Cell line**	**CDDP → triptolide**	**Triptolide → CDDP**	**CDDP → NSC130813**	**NSC130813 → CDDP**
U-2OS	ANT (2.14)	ANT (1.38)	ANT (1.67)	ANT (2.29)
U-2OS/CDDP300	ANT (1.38)	ANT (5.02)	ANT (1.96)	SYN (0.73)
U-2OS/CDDP4 μg	ANT (4.55)	ANT (2.23)	ANT (5.20)	ADD (1.09)
Saos-2	ADD (1.08)	ADD (0.99)	ANT (2.45)	SYN (0.73)
Saos-2/CDDP300	ANT (2.32)	ANT (2.17)	ANT (2.45)	ANT (1.86)
Saos-2/CDDP6 μg	ANT (9.19)	ANT (1.85)	ANT (9.87)	SYN (0.88)

*Legend: Data refer to at least two different determinations. Number in parenthesis indicate the combination index (CI) values. SYN, synergistic (CI < 0.90); ADD, additive (0.90 ≤ CI ≤ 1.10); ANT, antagonistic (CI > 1.10)*.

## Discussion

Many chemotherapeutic drugs, including several agents used in first-line and rescue chemotherapy protocols for OS exert their activity by directly or indirectly damaging DNA. Consequently, the ability of tumor cells to repair drug-induced DNA damages significantly impacts upon efficacy of these compounds (7, 8, 10, 11). Accordingly, the expression and activity of factors belonging to DNA repair pathways have been demonstrated to be involved in chemotherapy response and patients' outcome in different human tumors (7, 8, 12), with few findings also reported for OS (4, 9). This body of evidence has also indicated components related to DNA repair pathways as promising targets for innovative anticancer therapies, and several drugs interfering with these systems have entered phases I-II-III clinical trials (11, 17, 18).

Our study focused on a group of DNA repair genes and kinases, which we found to be upregulated in our panel of CDDP-resistant human OS cell lines, in order to verify whether they could be considered as new candidate therapeutic targets. In particular, analyses focused on genes belonging to the NER and BER pathways and on 18 druggable protein kinases, which resulted to be overexpressed in association with the development of CDDP resistance.

After a screening to identify the most effective siRNA to knock-down each prioritized gene, drug-sensitive and resistant cell lines were silenced and treated with CDDP, in order to identify those genes which down-regulation produced a corresponding increase of the *in vitro* CDDP sensitivity, confirming its involvement in reduced sensitivity to this drug. The genes that emerged to be most strictly related to CDDP unresponsiveness, were prioritized as candidate targets for the second phase of the study, in which their increased protein expression was confirmed in CDDP-resistant cells, justifying the subsequent *in vitro* studies of drugs interacting with these markers or their pathways.

The impact of these genes for DNA repair activity in our experimental models was further confirmed by functional analyses, in which the cells' capability to repair CDDP-induced DNA damages was assessed after silencing of each prioritized gene by the COMET assay (Supplementary Material). This evaluation showed that all these genes were, at different extent, significantly involved in this process since its knock-down produced a decrease of DNA repair activity in both sensitive and CDDP-resistant cell lines. These findings further support their value as candidate drug targets which may be considered for planning treatment strategies based on the synthetic lethality principle.

Drugs targeting the prioritized targets or pathways were selected on the basis of their reported promising activity in other experimental models and/or their use in clinical trials for human tumors. Among the 15 evaluated agents, TGX221, AZD6482, FR180204, AZD4547, GDC0994 and, at a lower extent, quercetin showed a reduced *in vitro* activity in CDDP-resistant variants compared to parental cell lines, suggesting the presence of cross-resistance mechanisms. The possible reasons for this apparent cross-resistance were not further explored because they were beyond the aims of this study. However, it can be hypothesized that cross-resistance might be due either to differential expression of transporters that recognize these drugs as substrate and efflux them out of the cells or to detoxification processes that are more active in CDDP resistant cells and inactivate these agents. Other reasons may be the activation of alternative or redundant pathways, which replace the function of the targeted pathway in CDDP resistant cells, which consequently become less sensitive to these drugs.

NSC130813 (NERI02; F06; targeting ERCC1 and ERCC4/XPF) and triptolide (targeting ERCC3/XPB) proved to be the two agents with the most relevant activity on both CDDP-sensitive and -resistant cell lines. Moreover, these two drugs did not show evidence of cross-resistance with CDDP and proved to reverse CDDP resistance in all drug-sensitive and -resistant cell lines. For these reasons, they were further tested in combined treatments (association and sequential exposure) with CDDP, in order to verify whether these combinations may lead to positive interactions.

When considered together, results obtained by the combined treatments indicated that NSC130813 (NERI02; F06) and triptolide have to be administered together with CDDP, in order to improve its efficacy in both drug-sensitive and resistant OS cells. If transferred to a clinical setting, this association has to be considered regarding possible effects of additive collateral toxicities.

NSC130813 (NERI02, F06), also known as [4-[(6-chloro-2-methoxy-9-acridinyl)amino]-2-[(4-methyl-1-piperazinyl)methyl]], is a compound which was shown to act synergistically with CDDP and mitomycin C by interfering DNA repair through the disruption of the interaction between ERCC1 and ERCC4/XPF (19). Targeting the ERCC1-ERCC4/XPF complex is an interesting approach to improve activity of DNA damaging drugs, because this complex plays a primary role in several DNA repair pathways, in addition to NER (19–21). The inhibition of ERCC1-ERCC4/XPF endonuclease activity is a relatively new strategy, which has been scarcely explored and for which no data have been reported yet for OS. Our study provided the proof-of-concept that targeting this complex may become an interesting future option also for OS treatment. Recent studies have provided important information that can be effectively used in the rational design of ERCC4/XPF inhibitors (10, 18, 20), which may therefore soon become available for clinical use.

Triptolide is a diterpene triepoxide isolated from a traditional Chinese medicinal plant with anti-inflammatory, immunosuppressive, contraceptive and antitumor activities (22). In the MG63 human OS cell line, triptolide proved to induce apoptosis and inhibit angiogenesis (23).

It has been demonstrated that triptolide covalently binds to human ERCC3/XPB, inhibiting its DNA repair-related activity (22, 24, 25). This ability to block DNA repair has important implications for the anticancer activity of CDDP, which effectiveness has been shown to be enhanced by the combined treatment with triptolide (26). In agreement with that, experimental studies confirmed that low concentrations of triptolide were able to potentiate the CDDP activity in human lung cancer (27) and human bladder CDDP-resistant cells (28).

On the basis of this body of evidence, we have explored whether in OS cells triptolide-mediated inhibition of NER may improve CDDP activity. Our findings indicated that inhibiting DNA repair through the simultaneous administration of CDDP and triptolide may be a new interesting treatment avenue to overcome CDDP resistance in OS.

In clinical setting, it is worthwhile noting that minnolide, a highly water-soluble analog of triptolide, has been recently included in trials for pancreatic cancer (ClinicalTrials.gov Identifier: NCT03117920), acute myeloid leukemia (ClinicalTrials.gov Identifier: NCT03347994), and different advanced solid tumors (ClinicalTrials.gov Identifier: NCT03129139), but results of these regimens are presently not available. Moreover, other triptolide derivatives and analogs have been used in clinical studies aimed to test their efficacy and safety (22).

Taken together these results indicated that targeting NER factors may have clinical relevance for OS treatment, with the hope that new drugs will become soon available, since few NER inhibitors have entered clinical trials so far.

In addition to DNA repair systems, different checkpoints may be induced by DNA damage to transiently delay or arrest cell cycle progression, providing time to the cell for repair before progressing into cell cycle or being addressed toward apoptosis (7, 11). Indeed, a variety of regulators including kinases, phosphatases, ubiquitin ligases, deubiquitinases, and other protein modifying enzymes, have been shown to modulate the activity and levels of key proteins belonging to different DNA repair pathways (13). In particular, protein kinases have been indicated to be involved or interfere with response to drug-induced DNA damages (13), despite their actual role in this process must be carefully investigated and validated inside each specific tumor type and only few preliminary information has been reported for OS so far (14, 15).

In this study, we have determined the *in vitro* activity of 13 drugs inhibiting kinases pathways and proteins, which resulted to be overexpressed in U-2OS- and/or Saos-2-derived CDDP-resistant variants compared to parental cells. Among the tested kinase targeting drugs, GDC0994 (targeting the MAPK pathway) and PD173074 (targeting FGFR1) showed some promising activity, without evidence of cross-resistance with CDDP. Although these drugs were not further analyzed in combined treatments with CDDP, the obtained findings suggest that targeting protein kinases that influence DNA repair activities may indicate new promising therapeutic perspectives in OS, demanding for additional investigation. This perspective is particularly interesting because there are many protein kinase inhibitors in various stages of clinical development worldwide and the majority of them are used for cancer treatment (29).

Our results can also be the basis for further *in vitro* and *in vivo* studies aimed to improve the translation of these finding into the clinic. Development of 3D *in vitro* models may provide additional insights about the efficacy of these drugs against a tumor mass. Assessment of the efficacy of these agents in patient derived xenograft (PDX) models may further support their clinical use. All these activities are presently planned and will focus on the drugs screened and highlighted by this study.

## Conclusions

In high-grade OS, when patients fail to respond to first-line treatment and relapse, therapeutic options and drugs effective for rescue chemotherapy protocols are scarce, also because resistance mechanisms developed against first-line chemotherapeutic drugs can also be responsible for reduced responsiveness to the agents used in the subsequent regimens.

Inhibition of DNA repair can be considered as a promising treatment strategy to enhance the efficacy of currently available DNA damaging drugs.

There are several genes and proteins involved in modulating the cellular response to DNA damage, each one may serve as target to enhance the efficacy of conventional therapeutic modalities. The current efforts in the development and deployment of several classes of DNA repair targeting compounds justify the hope to achieve new tailored treatment approaches through the use of these inhibitor drugs, which may ultimately drive toward innovative regimens aimed to improve patient outcomes.

The evidence emerged within this study about the possibility of successfully combining CDDP with drugs targeting DNA repair factors or protein kinases involved in these processes may indeed indicate new therapeutic options for specific OS patient cohorts, who have reduced cure probabilities.

## Data Availability Statement

Datasets generated and analyzed in this study will be made available by the authors to qualified researchers, upon justified request.

## Author Contributions

ET, MF, CH, and MS designed the study. MS coordinated the study and drafted the manuscript, which was revised by all authors, who approved it for publication. ET, MF, AF-R, MP, and FM carried out the cell culture-based experiments and the *in vitro* drug testing. ET, MF, SV, and AF-R performed the gene silencing screening. ET and MF carried out the DNA damage evaluations. ET, MF, CH, and SL took part in the molecular biology and western blot studies. ET, MF, CH, MP, and MS performed the statistical analyses. All authors have made a substantial, direct, and intellectual contribution to the work.

### Conflict of Interest

The authors declare that the research was conducted in the absence of any commercial or financial relationships that could be construed as a potential conflict of interest. CH is Associate Editor of the section Pharmacogenetics and Pharmacogenomics of Frontiers in Pharmacology.

## Abbreviations

ADD: additive
ANT: antagonistic
BER: base excision repair
CDDP: cisplatin
CDKs: cyclin-dependent kinases
CI: combination index
MTT: 3-(4,5-dimethylthiazol-2-yl)-2,5-dephenyltetrazolium bromide
NER: nucleotide excision repair
OS: osteosarcoma
PDX: patient derived xenograft
qRT-PCR: quantitative reverse transcriptase-polymerase chain reaction
SYN: synergistic
TBST: Tris-Buffered Saline and Tween 20.
