# Supplementary material for: Cisplatin Resistance in Osteosarcoma: In vitro Validation of Candidate DNA Repair-Related Therapeutic Targets and Drugs for Tailored Treatments
Source: Front Oncol. 2020 Mar 10;10:331. doi: 10.3389/fonc.2020.00331 (PMC7077033; doi:10.3389/fonc.2020.00331)
Supplement: Supplementary file 1 [file Table_1.DOC]

**Supplementary Table 1.** Assays used to evaluate gene expression levels by quantitative RT-PCR in human osteosarcoma cell lines.

| **Gene Name (Entrez-Gene ID)** | **TaqMan Gene Expression Assay ID** |
| --- | --- |
| *ERCC1* (2067) | Hs01012158_m1 |
| *ERCC2/XPD* (2068) | Hs00361161_m1 |
| *ERCC3/XPB* (2071) | Hs01554457_m1 |
| *ERCC4/XPF* (2072) | Hs00193342_m1 |
| *ERCC5/XPG* (2073) | Hs01557031_m1 |
| *XPA* (7507) | Hs00166045_m1 |
| *PARP1* (142) | Hs00242302_m1 |
| *PARP2* (10038) | Hs00193931_m1 |
| *AKT3* (10000) | Hs00987350_m1 |
| *CDK3* (1018) | Hs00176202_m1 |
| *CDK6* (1021) | Hs01026371_m1 |
| *CDK8* (1024) | Hs00176209_m1 |
| *CDK9* (1025) | Hs00977896_g1 |
| *CDK10* (8558) | Hs00177586_m1 |
| *FGFR1* (2260) | Hs00915142_m1 |
| *FGFR2* (2263) | Hs01552918_m1 |
| *FLT4* (2324) | Hs01047677_m1 |
| *MAP2K2* (5605) | Hs00360961_m1 |
| *MAP2K3* (5606) | Hs00177127_m1 |
| *MAP2K5* (5607) | Hs00177134_m1 |
| *MAP2K7* (5609) | Hs00178198_m1 |
| *MAPK1* (5594) | Hs01046830_m1 |
| *MAPK3* (5595) | Hs00946872_m1 |
| *PIK3C2A* (5286) | Hs00904054_m1 |
| *PIK3C3* (5289) | Hs00176908_m1 |
| *PIK3CB* (5291) | Hs00927728_m1 |
