# Supplementary material for: Cisplatin Resistance in Osteosarcoma: In vitro Validation of Candidate DNA Repair-Related Therapeutic Targets and Drugs for Tailored Treatments
Source: Front Oncol. 2020 Mar 10;10:331. doi: 10.3389/fonc.2020.00331 (PMC7077033; doi:10.3389/fonc.2020.00331)
Supplement: Supplementary file 2 [file Table_2.DOC]

**Supplementary Table 2.** Antibodies used to evaluate gene expression by western blot in human osteosarcoma cell lines.

| **Gene Name (UniProt ID)** | **Antibody name (clone, company): dilution rate** |
| --- | --- |
| *ERCC1* (2067) | FL-297 rabbit pAb (sc-10785, Santa Cruz Biotechnology, Inc); 1:200  8F1 mouse mAb (sc-56673, Santa Cruz Biotechnology, Inc); 1:500 |
| *ERCC2/XPD* (2068) | TFIIH p80 (H-150) rabbit (sc-20696, Santa Cruz Biotechnology, Inc); 1:200 (against amino acids 611-760) |
| *ERCC3/XPB* (19447) | 2C6H6G5 mouse mAb (#8746, Cell Signaling Technology); 1:1000 |
| *ERCC4/XPF* (Q92889) | 3F2/3 mouse mAb (sc-136153, Santa Cruz Biotechnology, Inc); 1:200 (against amino acids 629-905) |
| *XPA* (P23025) | B-1 mouse mAb (sc-2853, Santa Cruz Biotechnology, Inc); 1:500 (against amino acids 1-273, representing full length protein) |
| *FGFR1*  (Swiss-Prot Acc. P11362) | D8E4 XP, rabbit mAb (#9740, Cell Signaling Technology); 1:1000 |
| *MAP2K3*  (Swiss-Prot Acc. P46734) | MKK3 rabbit pAb (#5674, Cell Signaling Technology); 1:1000 |
| *MAP2K7*  (Swiss-Prot Acc. O14733) | MKK7 rabbit pAb (#4172, Cell Signaling Technology); 1:1000 |
| *MAPK3* (5595), p42/p44  (Swiss-Prot Acc. P27361 e P28482) | p44/42 MAPK (Erk1/2), 137F5 rabbit mAb (#4695, Cell Signaling Technology); 1:1000 |
| *PIK3CB* (P42338) | PI3 Kinase p110beta, rabbit mAb (C33D4) (#3011, Cell Signaling Technology); 1:1000 |
