# Supplementary material for: Cisplatin Resistance in Osteosarcoma: In vitro Validation of Candidate DNA Repair-Related Therapeutic Targets and Drugs for Tailored Treatments
Source: Front Oncol. 2020 Mar 10;10:331. doi: 10.3389/fonc.2020.00331 (PMC7077033; doi:10.3389/fonc.2020.00331)
Supplement: Supplementary file 3 [file Table_3.DOC]

**SUPPLEMENTARY Table 3.** Drugs targeting the genes/pathways prioritized by the gene silencing approaches, which have been tested in this study. For each drug, only active or completed clinical trials involving cancer patients are listed. Trials without any information about recruitment *status* or activity, as well as trials which were suspended or terminated, have not been considered. Each trial has been indetified with its corresponding ClinicalTrials.gov identifier (NCT number). Information are updated to September 30th, 2019.

| ***Drug*** | ***Target(s)*** | ***Mechanism of action*** | ***Phase of clinical development in cancer*** |
| --- | --- | --- | --- |
| NSC130813 (NERI02, F06) | ERCC1; ERCC4/XPF | Inhibition of the interactions between ERCC1 and ERCC4/XPF leading to a decrease of NER-mediated repair of DNA damages | No active or completed clinical trials in human cancer patients. |
| Triptolide | ERCC3/XPB | Covalent binding to human ERCC3/XPB and inhibition of its DNA repair-related activity | No active or completed clinical trials in human cancer patients. |
| X80 | XPA | Reduction of XPA-DNA complex formation through a direct X80-XPA interaction | No active or completed clinical trials in human cancer patients. |
| AZD6482  GSK2636771  Quercetin  TGX221 | PIK3 kinases | Inhibition of PI3K kinases activity | No active or completed clinical trials in human cancer patients for AZD6482, Quercetin, and TGX221.  GSK2636771:  - A Phase I/IIa, first time in human, study of GSK2636771 in patients with advanced solid tumors with phosphatase and tensin homolog (PTEN) deficiency (NCT01458067 - completed; no results posted)  - Phase I study in which GSK2636771 is given in combination with pembrolizumab to control the disease in patients with refractory (has not responded to treatment) metastatic melanoma and PTEN loss (NCT03131908 - recruiting)  - Phase I, open-label, dose-finding, multicenter study designed to determine the recommended Phase II dose (RP2D) for the combination of GSK2636771 with enzalutamide in male subjects with metastatic castration-resistant prostate cancer (NCT02215096 - active, not recruiting)  - Phase Ib/IIa, open-label, non-randomized, dose-escalation, multi-center study to evaluate the safety, tolerability, pharmacokinetics, and clinical activity of oral GSK2636771 in combination with paclitaxel in advanced gastric adenocarcinoma patients with PTEN-deficiency (NCT02615730 - recruiting)  - Phase II treatment directed by genetic testing in patients with solid tumors or lymphomas that have progressed following at least one line of standard treatment or for which no agreement upon treatment approach exists (NCT02465060- recruiting) |
| FR180204  GDC0994  Ulixertinib  (BVD-523) | MAPK1-3 | Inhibitors of MAPK signaling-regulated kinases | No active or completed clinical trials in human cancer patients for FR180204.  GDC-0994:  - Phase I, open-label, multicenter, dose-escalation study to assess the safety, tolerability, and pharmacokinetics of GDC-0994 in patients with locally advanced or metastatic solid tumors (NCT01875705 - completed; no results posted)  - Phase I, dose-escalation study to assess the safety, tolerability and effects of oral dosing of GDC-0994 administered in combination with cobimetinib in patients with histologically confirmed, locally advanced, or metastatic solid tumors for which standard therapies either do not exist or have proven ineffective or intolerable (NCT02457793 - completed, with posted results)  ULIXERTINIB (BVD-523):  - Phase I trial in combination with the CDK4/6 inhibitor palbociclib in patients with advanced pancreatic and other solid tumors (NCT03454035 - recruiting)  - Phase I study in combination with nab-paclitaxel plus gemcitabine in patients with newly diagnosed metastatic pancreatic cancer (NCT02608229 - active, not recruiting)  - Phase I/II trial in patients with advanced malignancies (NCT01781429 - completed; no results posted)  - Phase I/II trial in patients with acute myelogenous leukemia or myelodysplastic syndrome (NCT02296242 - completed, with posted results)  - Phase II trial in patients with advanced uveal melanoma (NCT03417739 - active, not recruiting)  - Phase II treatment directed by genetic testing in patients with pediatric patients with solid tumors, non-Hodgkin lymphomas, or histiocytic disorders that have progressed following at least one line of standard systemic therapy and/or for which no standard treatment exists that has been shown to prolong survival (NCT03155620 - recruiting)  - Phase II treatment directed by genetic testing in patients with solid tumors or lymphomas that have progressed following at least one line of standard treatment or for which no agreement upon treatment approach exists (NCT02465060 - recruiting) |
| Hypothemycin | MAP2K3; MAP2K7 | Inhibition of MAPK kinases pathways | No active or completed clinical trials in human cancer patients. |
| AZD4547  FIIN-2  PD173074  SSR128129E | FGFR1 | PD173074:  ATP-competitive inhibitor of FGFR1 and inhibition of FGFR1 autophosphorylation  AZD4547, FIIN-2, SSR128129E:  FGFRs irreversible inhibition | No active or completed clinical trials in human cancer patients for FIIN-2, PD173074, and SSR128129E.  AZD4547:  - Phase I/II patients with hormone sensitive (oestrogen receptor positive) breast cancer, who have progressed on treatment with either anastrozole or letrozole (NCT01791985; completed; no results posted)  - Phase I/II in association with docetaxel in patients with recurrent non-small cell lung cancer (NCT01824901- completed, with posted results)  - Phase II/III trial for previously treated patients with stage IV squamous cell lung cancer (NCT02965378 - active, not recruiting)  - Phase I/II trial to assess the safety and effectiveness of AZD4547 in combination with fulvestrant vs. fulvestrant alone in ER+ breast cancer patients with FGFR1 polysomy (FISH4/5) or gene amplification (NCT01202591 - completed, with posted results)  - Phase I study is to explore the safety and tolerability of AZD4547 in Japanese patients with advanced solid malignancies (NCT01213160 - completed, with posted results)  - Phase 1b study in patients with muscle invasive bladder cancer (MIBC) (urothelial) who have progressed on prior treatment (NCT02546661 - active, not recruiting)  - Phase II study consisting of a series of parallel multi-centre single arms, each testing an experimental targeted drug in a population stratified by multiple pre-specified actionable target putative biomarkers (NCT02664935 - recruiting)  - Phase II treatment directed by genetic testing in patients with solid tumors or lymphomas that have progressed following at least one line of standard treatment or for which no agreement upon treatment approach exists (NCT02465060- recruiting)  - Phase I/II study in patients with recurrent IDH wild-type gliomas with FGFR3-TACC3 or FGFR1-TACC1 fusion (NCT02824133 - completed; no results posted) |

**References cited in the Table**

1. Jordheim LP, Barakat KH, Heinrich-Balard L, Matera EL, Cros-Perrial E, Bouledrak K, et al. Small molecule inhibitors of ERCC1-XPF protein-protein interaction synergize alkylating agents in cancer cells. *Mol Pharmacol* (2013) 84(1):12-24. Epub 2013/04/13. doi: 10.1124/mol.112.082347

mol.112.082347 [pii]. PubMed PMID: 23580445.

2. He QL, Titov DV, Li J, Tan M, Ye Z, Zhao Y, et al. Covalent modification of a cysteine residue in the XPB subunit of the general transcription factor TFIIH through single epoxide cleavage of the transcription inhibitor triptolide. *Angew Chem Int Ed Engl* (2015) 54(6):1859-63. Epub 2014/12/17. doi: 10.1002/anie.201408817. PubMed PMID: 25504624; PubMed Central PMCID: PMC4314353.

3. Titov DV, Gilman B, He QL, Bhat S, Low WK, Dang Y, et al. XPB, a subunit of TFIIH, is a target of the natural product triptolide. *Nat Chem Biol* (2011) 7(3):182-8. Epub 2011/02/01. doi: 10.1038/nchembio.522

nchembio.522 [pii]. PubMed PMID: 21278739; PubMed Central PMCID: PMC3622543.

4. Neher TM, Shuck SC, Liu JY, Zhang JT, Turchi JJ. Identification of novel small molecule inhibitors of the XPA protein using in silico based screening. *ACS Chem Biol* (2010) 5(10):953-65. Epub 2010/07/29. doi: 10.1021/cb1000444. PubMed PMID: 20662484; PubMed Central PMCID: PMC2955790.

5. Safdari Y, Khalili M, Ebrahimzadeh MA, Yazdani Y, Farajnia S. Natural inhibitors of PI3K/AKT signaling in breast cancer: emphasis on newly-discovered molecular mechanisms of action. *Pharmacol Res* (2015) 93:1-10. Epub 2014/12/24. doi: 10.1016/j.phrs.2014.12.004

S1043-6618(14)00190-X [pii]. PubMed PMID: 25533812.

6. Varghese E, Samuel SM, Abotaleb M, Cheema S, Mamtani R, Busselberg D. The "Yin and Yang" of Natural Compounds in Anticancer Therapy of Triple-Negative Breast Cancers. *Cancers (Basel)* (2018) 10(10). Epub 2018/09/27. doi: E346 [pii]

10.3390/cancers10100346

cancers10100346 [pii]. PubMed PMID: 30248941; PubMed Central PMCID: PMC6209965.

7. Wang X, Ding J, Meng LH. PI3K isoform-selective inhibitors: next-generation targeted cancer therapies. *Acta Pharmacol Sin* (2015) 36(10):1170-6. Epub 2015/09/15. doi: 10.1038/aps.2015.71

aps201571 [pii]. PubMed PMID: 26364801; PubMed Central PMCID: PMC4648175.

8. Patnaik S, Dietz HC, Zheng W, Austin C, Marugan JJ. Multi-gram scale synthesis of FR180204. *J Org Chem* (2009) 74(22):8870-3. Epub 2009/10/27. doi: 10.1021/jo901835m. PubMed PMID: 19852504; PubMed Central PMCID: PMC2862695.

9. Uehling DE, Harris PA. Recent progress on MAP kinase pathway inhibitors. *Bioorg Med Chem Lett* (2015) 25(19):4047-56. Epub 2015/08/25. doi: 10.1016/j.bmcl.2015.07.093

S0960-894X(15)00812-4 [pii]. PubMed PMID: 26298497.

10. Fukazawa H, Ikeda Y, Fukuyama M, Suzuki T, Hori H, Okuda T, et al. The resorcylic acid lactone hypothemycin selectively inhibits the mitogen-activated protein kinase kinase-extracellular signal-regulated kinase pathway in cells. *Biol Pharm Bull* (2010) 33(2):168-73. Epub 2010/02/02. doi: JST.JSTAGE/bpb/33.168 [pii]

10.1248/bpb.33.168. PubMed PMID: 20118535.

11. Schirmer A, Kennedy J, Murli S, Reid R, Santi DV. Targeted covalent inactivation of protein kinases by resorcylic acid lactone polyketides. *Proc Natl Acad Sci U S A* (2006) 103(11):4234-9. Epub 2006/03/16. doi: 0600445103 [pii]

10.1073/pnas.0600445103. PubMed PMID: 16537514; PubMed Central PMCID: PMC1449676.

12. Kumar SB, Narasu L, Gundla R, Dayam R, J ARPS. Fibroblast growth factor receptor inhibitors. *Curr Pharm Des* (2013) 19(4):687-701. Epub 2012/09/29. doi: CPD-EPUB-20120926-14 [pii]. PubMed PMID: 23016864.

13. Herbert C, Schieborr U, Saxena K, Juraszek J, De Smet F, Alcouffe C, et al. Molecular mechanism of SSR128129E, an extracellularly acting, small-molecule, allosteric inhibitor of FGF receptor signaling. *Cancer Cell* (2013) 23(4):489-501. Epub 2013/04/20. doi: 10.1016/j.ccr.2013.02.018

S1535-6108(13)00074-3 [pii]. PubMed PMID: 23597563.

14. Hierro C, Rodon J, Tabernero J. Fibroblast Growth Factor (FGF) Receptor/FGF Inhibitors: Novel Targets and Strategies for Optimization of Response of Solid Tumors. *Semin Oncol* (2015) 42(6):801-19. Epub 2015/11/29. doi: 10.1053/j.seminoncol.2015.09.027

S0093-7754(15)00197-9 [pii]. PubMed PMID: 26615127.

15. Tan L, Wang J, Tanizaki J, Huang Z, Aref AR, Rusan M, et al. Development of covalent inhibitors that can overcome resistance to first-generation FGFR kinase inhibitors. *Proc Natl Acad Sci U S A* (2014) 111(45):E4869-77. Epub 2014/10/29. doi: 10.1073/pnas.1403438111

1403438111 [pii]. PubMed PMID: 25349422; PubMed Central PMCID: PMC4234547.

16. Saka H, Kitagawa C, Kogure Y, Takahashi Y, Fujikawa K, Sagawa T, et al. Safety, tolerability and pharmacokinetics of the fibroblast growth factor receptor inhibitor AZD4547 in Japanese patients with advanced solid tumours: a Phase I study. *Invest New Drugs* (2017) 35(4):451-62. Epub 2017/01/11. doi: 10.1007/s10637-016-0416-x

10.1007/s10637-016-0416-x [pii]. PubMed PMID: 28070720; PubMed Central PMCID: PMC5502072.
