# Supplementary material for: Cisplatin Resistance in Osteosarcoma: In vitro Validation of Candidate DNA Repair-Related Therapeutic Targets and Drugs for Tailored Treatments
Source: Front Oncol. 2020 Mar 10;10:331. doi: 10.3389/fonc.2020.00331 (PMC7077033; doi:10.3389/fonc.2020.00331)
Supplement: Supplementary file 4 [file Table_4.DOC]

**Supplementary Table 4.** Most active siRNAs selected from a screening of three different siRNAs for each gene.

| **Gene** | **siRNA ID** |
| --- | --- |
| *ERCC1* | s4785 |
| *ERCC2/XPD* | s4789 |
| *ERCC3/XPB* | s4797 |
| *ERCC4/XPF* | s4801 |
| *ERCC5/XPG* | s4803 |
| *XPA* | s14927 |
| *PARP1* | s1097 |
| *PARP2* | s19504 |
| *AKT3* | s19428 |
| *CDK3* | s2819 |
| *CDK6* | s1021 |
| *CDK8* | s2832 |
| *CDK9* | s2834 |
| *CDK10* | s264 |
| *FGFR1* | s5166 |
| *FGFR2* | s5175 |
| *FLT4* | s5295 |
| *MAP2K2* | s11172 |
| *MAP2K3* | s57134 |
| *MAP2K5* | s11178 |
| *MAP2K7* | s11183 |
| *MAPK1* | s11137 |
| *MAPK3* | s230180 |
| *PIK3C2A* | s10509 |
| *PIK3C3* | s10517 |
| *PIK3CB* | s10525 |
