# Supplementary material for: Cisplatin Resistance in Osteosarcoma: In vitro Validation of Candidate DNA Repair-Related Therapeutic Targets and Drugs for Tailored Treatments
Source: Front Oncol. 2020 Mar 10;10:331. doi: 10.3389/fonc.2020.00331 (PMC7077033; doi:10.3389/fonc.2020.00331)
Supplement: Supplementary file 5 [file Data_Sheet_1.PDF]

Supplementary Material. *Effects of gene silencing on DNA repair activity after treatment with cisplatin (CDDP), as estimated with the alkaline comet assay.*

Materials and Methods

Effects of gene silencing on DNA repair activity after treatment with CDDP were estimated with the alkaline comet assay. Time-course setting experiments were performed to determine the time-points at which the treatment with CDDP produced the most extensive DNA damage. According to the evidence collected in the setting experiments, the U-2OS cells were treated with CDDP 10µM for 30 min, whereas its CDDP-resistant variants (U-2OS/CDDP300; U-2OS/CDDP4µg) were treated with CDDP 50µM for 30 min. The impact of gene silencing on DNA repair activity was assessed immediately, one and three hours after the end of CDDP treatment.

The alkaline comet assay was performed using the Trevigen's CometAssay® single cell gel electrophoresis kit (Trevigen, Gaithersburg, MD) following the manufacturer's instructions. Briefly, after CDDP treatment, cells were harvested and counted with the Trypan Blue dye exclusion method. A volume containing 1×10^4 cells was added to 200µL of molten LMAgarose (0.5% low-melting agarose) kept at 38°C. After mixing the sample, a 25µL aliquot was pipetted onto an area of the CometSlide. The slide was incubated at 4°C for 10 min to accelerate gelling of the agarose disc and then transferred to prechilled lysis solution for 30 min at 4°C. A denaturation step was performed in unwinding solution, pH>13 (300 mM NaOH, 1 mM EDTA), at room temperature (RT) for 30 min, shielded from light. The slide was then transferred in a horizontal chamber in fresh running buffer, pH >13 (200 mM NaOH, 1 mM EDTA), for electrophoresis at 21 Volts for 30 min. The slide was fixed in 70% ethanol at RT for 5 min and dried. For observation, samples were stained with SYBR® Gold nucleic acid stain 10,000 concentrate in DMSO (S11494, Invitrogen European Headquaters, Paisley, UK), diluted 1:10 000 in PBS 1X and observed by a fluorescence microscope (Nikon Eclipse 90i, Chiyoda-Tokyo, Japan). For each experimental point, images of at least 100 cells were collected and comet tails (cells with residual DNA damages) were analyzed and quantified with the Comet Analysis Software (Trevigen). Since the relative length and intensity of DNA tails to heads is proportional to the amount of DNA damage present in each individual nucleus, the amount of DNA breaks was quantified by calculating the tail moment (TM), which is the product of the tail length and the fraction of total DNA present in the tail. The significance of difference between the mean TM of controls (non-silenced) and silenced cells was estimated by using the two-tailed Mann Whitney U-test.

RESULTS

Results of a representative experiment are summarized in **Supplementary Table 5**.

Decrease of DNA repair activity of CDDP-induced damages after silencing of the prioritized candidate drug targets was assessed with the alkaline comet assay on the U-2OS cell line and its variants with the lowest (U-2OS/CDDP300) and highest (U-2OS/CDDP4µg) CDDP resistance levels. The significant increase of TM in silenced cells compared to controls was used to indicate a significant decrease in DNA repair activity as consequence to gene knock-down.

The results obtained after silencing of NER genes *(ERCC1, ERCC2/XPD, ERCC3/XPB, ERCC4/XPF, XPA)* can be summarized as follows:

i) in U-2OS parental cells, silencing of each NER gene produced a significant decrease of DNA damages repair activity, which was more evident at one and three hours after the end of CDDP treatment

ii) in U-2OS/CDDP300 cells, a significant decrease of DNA damages repair activity after gene knock-down was almost invariably found, with the only exception for silencing of *ERCC3/XPB*

iii) in U-2OS/CDDP4µg cells, the most relevant effects on DNA damages repair activity emerged three hours after the end of CDDP treatment (with the only exception for silencing of *ERCC3/XPB*, which knock-down produced the most evident effect one hour after the end of CDDP treatment).

The results obtained after silencing of kinase genes *(MAPK3, MAP2K3, MAP2K7, FGFR1, PIK3CB)* can be summarized as follows:

i) in U-2OS parental cells, silencing of kinase genes produced a significant decrease of DNA damages repair activity detected three hours after the end of CDDP treatment (with the only exception for silencing of *MAP2K3*, which knock-down produced the most evident effect immediately after the end of CDDP treatment)

ii) in U-2OS/CDDP300 cells, a significant decrease of DNA damages repair activity was observed after silencing of *MAP2K7, FGFR1, PIK3CB* genes, especially at three hours after the end of CDDP treatment

iii) in U-2OS/CDDP4μg cells, a significant decrease of DNA damages repair activity was observed after silencing of each kinase gene either immediately and/or at three hours after the end of CDDP treatment.

Considering all the data obtained by these experiments, the involvement in DNA repair activity of prioritized genes, and thus their potential value as therapeutic targets, were further supported.

Supplementary Table 5. Effects of gene silencing on DNA repair activity after treatment with CDDP, as estimated with the alkaline comet assay. The impact of gene silencing on DNA repair activity was assessed immediately at the end of CDDP treatment, one and three hours after the end of CDDP treatment. Numbers refer to tail moment values derived from one representative experiment. P values indicate the significance of difference between silenced cells and the non-silenced controls, as determined by using the two tailed Mann-Whitney U-Test (only significant P values are listed and highlighted).

Cell line	***Gene***	***DNA repair activity immediately after treatment with CDDP***			***DNA repair activity 1 hour after the end of CDDP treatment***			***DNA repair activity 3 hours after the end of CDDP treatment***		
U-2OS		*Control*	*Silenced cells*	*P*	*Control*	*Silenced cells*	*P*	*Control*	*Silenced cells*	*P*
	NER pathway									
	ERCC1	1.51	4.72	< 0.001	0.52	4.45	< 0.001	0.26	1.84	< 0.001
	ERCC2/XPD	5.11	4.38		3.25	6.81	< 0.001	2.21	4.82	< 0.001
	ERCC3/XPB	0.62	1.09	< 0.01	0.78	1.51	< 0.001	0.73	0.98	
	ERCC4/XPF	1.51	1.65		0.52	3.34	< 0.001	0.26	1.71	< 0.001
	XPA	1.51	3.05		0.52	1.72	< 0.001	0.26	1.38	< 0.01
	Kinases									
	MAPK3	0.62	1.36		0.78	1.42		0.73	2.37	< 0.01
	MAP2K3	0.62	1.91	< 0.01	0.78	2.10		0.73	1.08	
	MAP2K7	5.11	2.51		3.25	2.42		2.21	3.31	< 0.05
	FGFR1	5.11	4.60		3.25	3.63		2.21	3.04	< 0.05
	PIK3CB	5.11	5.62		3.25	3.16		2.21	3.50	< 0.05

Supplementary Table 5. *(continued)*

Cell line	*Gene*	*DNA repair activity immediately after treatment with CDDP*			*DNA repair activity 1 hour after the end of CDDP treatment*			*DNA repair activity 3 hours after the end of CDDP treatment*		
U-2OS/CDDP300		*Control*	*Silenced cells*	*P*	*Control*	*Silenced cells*	*P*	*Control*	*Silenced cells*	*P*
	NER pathway									
	ERCC1	3.36	5.73	< 0.001	3.16	5.59	< 0.01	1.98	4.39	< 0.001
	ERCC2/XPD	1.61	3.26	< 0.01	1.77	2.55		1.16	2.81	< 0.001
	ERCC3/XPB	3.76	3.47		1.98	4.24		1.57	2.27	
	ERCC4/XPF	1.60	3.19	< 0.01	3.16	6.23	< 0.001	1.98	5.94	< 0.001
	XPA	3.35	6.70	< 0.001	3.16	6.34	< 0.001	1.98	7.28	< 0.001
	Kinases									
	MAPK3	3.76	3.75		1.98	4.57		1.57	5.56	
	MAP2K3	3.76	9.02		1.98	3.32		1.57	2.67	
	MAP2K7	3.76	3.33		1.98	3.31		1.57	2.51	< 0.05
	FGFR1	3.35	3.95		3.16	3.67		1.98	4.86	< 0.01
	PIK3CB	3.35	6.09	< 0.01	3.16	4.53	< 0.05	1.98	5.07	< 0.01

Supplementary Table 5. *(continued)*

Cell line	***Gene***	***DNA repair activity immediately after treatment with CDDP***			***DNA repair activity 1 hour after the end of CDDP treatment***			***DNA repair activity 3 hours after the end of CDDP treatment***		
U-2OS/CDDP4μg		*Control*	*Silenced cells*	*P*	*Control*	*Silenced cells*	*P*	*Control*	*Silenced cells*	*P*
	NER pathway									
	ERCC1	0.84	1.63	< 0.001	0.99	0.91		0.79	1.44	< 0.001
	ERCC2/XPD	0.84	1.32	< 0.01	0.99	1.40		0.79	1.63	< 0.001
	ERCC3/XPB	0.84	0.79		0.99	2.30	< 0.001	0.79	0.74	
	ERCC4/XPF	0.84	1.35	< 0.01	0.99	0.86		0.79	2.20	< 0.001
	XPA	0.84	0.98		0.99	1.06		0.79	1.24	< 0.05
	Kinases									
	MAPK3	0.84	1.01		0.99	1.02		0.79	1.34	< 0.01
	MAP2K3	0.84	1.18	< 0.01	0.99	0.67		0.79	1.02	< 0.05
	MAP2K7	0.84	1.30	< 0.01	0.99	0.98		0.79	1.35	< 0.01
	FGFR1	2.64	4.19	< 0.05	2.18	2.99	< 0.05	2.16	3.22	< 0.01
	PIK3CB	2.64	4.72	< 0.01	2.18	3.22	< 0.01	2.16	3.44	< 0.01
